# The Burden of Obesity in Egypt

**DOI:** 10.3389/fpubh.2021.718978

**Published:** 2021-08-27

**Authors:** Mohamed Aboulghate, Aliaa Elaghoury, Ibrahim Elebrashy, Nabil Elkafrawy, Galal Elshishiney, Ehab Abul-Magd, Engy Bassiouny, Dalia Toaima, Baher Elezbawy, Ahmad Fasseeh, Sherif Abaza, Zoltán Vokó

**Affiliations:** ^1^Department of Anesthesiology, Cairo University, Cairo, Budapest, Egypt; ^2^Department of Endocrinology, Alexandria University, Alexandria, Egypt; ^3^Department of Internal Medicine, Cairo University, Cairo, Egypt; ^4^Department of Internal Medicine, Endocrinology Unit, Menofia University, Menofia, Egypt; ^5^Ministry of Health and Population, Cairo, Egypt; ^6^Egyptian Health Care Management Society, Cairo, Egypt; ^7^Novo Nordisk, Cairo, Egypt; ^8^Syreon Middle East, Alexandria, Egypt; ^9^Department of Social Sciences, Eötvös Loránd University, Budapest, Hungary; ^10^Center for Health Technology Assessment, Semmelweis University, Budapest, Hungary; ^11^Syreon Research Institute, Budapest, Hungary

**Keywords:** cost analysis, epidemiology, health policy, obesity, mortality

## Abstract

**Objective:** Estimating the burden of obesity to society is an essential step in setting priorities and raising awareness. We aimed to assess the clinical, humanistic and economic burden of obesity for adults in Egypt.

**Methods:** We used the population attributable fraction concept to estimate the burden. A non-systematic review was conducted to estimate the prevalence of obesity and its comorbidities in addition to the obesity attributable fraction. Patient numbers, direct healthcare costs, disability adjusted life years (DALYs) and attributable mortality were estimated.

**Results:** Obesity is a major contributor to the development of diabetes mellitus, hypertension, obstructive sleep apnea and fatty liver, in addition to several serious diseases. The estimated annual deaths due to obesity was about 115 thousand (19.08% of the total estimated deaths in 2020). DALYs attributable to obesity may have reached 4 million in 2020.The economic burden imposed by obesity is around 62 Billion Egyptian pounds annually. This value is the cost of treating diseases attributable to obesity in adults.

**Conclusions:** Diseases attributable to obesity create a huge economic, humanistic, and clinical burden in Egypt. Reducing obesity could help dramatically decrease the catastrophic health effect of these diseases which in turn decreases mortality and DALYs lost.

## Introduction

Annually, about 4.7 million premature deaths occur due to obesity. It was ranked fifth among the leading preventable causes of death (1), making up 8.4% of deaths worldwide in 2017 (2).

According to the World Health Organization (WHO), Egypt ranks 18^th^ with the highest prevalence of obesity worldwide (3). Deaths attributable to non-communicable diseases represent about 71% of the total mortality burden (4).

Very few studies have been published about the burden of diseases in Egypt in general, and the burden of obesity is even more complex as the impact of obesity is a result of its comorbidities (5) rather than a direct effect, which makes it more difficult to estimate the burden of obesity. There is not any published study which has specifically attempted to evaluate the economic burden of obesity in Egypt.

Estimating the societal burden of obesity is an essential step in setting priorities for research and public health interventions. It also helps to raise public awareness about the negative impacts of obesity and provides health policy decision-makers with information about the magnitude of health problems (6). Some studies do not consider obesity as a disease, as it may not have substantial direct costs or related pain (7), although it is included in the International Classification of Diseases and Related Health Problems (8). The clear fact is that obesity is a risk factor for several costly and disabling diseases (7).

Our objective was to estimate the burden of obesity in Egypt. This study evaluates the clinical, humanistic, and economic burden of obesity in adults in Egypt.

## Materials and Methods

We utilized both primary (expert interviews) and secondary (publications and payers' services list) data sources to estimate the annual cost per patient per year for each comorbidity. For the considered diseases, we calculated the number of patients attributable to obesity using the population attributable fraction concept. We also estimated the annual economic burden, mortalities, and disability adjusted life years (DALYs). To estimate the prevalence of obesity among the adult population in Egypt, we used the results of 100 million health survey conducted in 2019 (unpublished data from personal communication). Obesity was defined as having a body mass index (BMI) ≥ 30 throughout this study based on WHO definition (9).

### Diseases Attributable to Obesity

To identify the diseases attributable to obesity, a non-systematic review was conducted through PubMed, Google and Google Scholar search engines. The search was conducted on 16^th^ of April 2020 with no time restriction. The search key can be found in Supplementary Table 1. We included studies that list the comorbidities associated with obesity. Finally, a primary list was based on five studies. This was followed by expert interviews to validate the included diseases. Finally, 13 diseases were included in the analysis: type 2 diabetes mellitus, hypertension, ischemic heart disease, hyperlipidemia, stroke, sleep apnea, osteoarthritis, gall bladder disease, colon and colorectal cancer, breast cancer, endometrial cancer, depression and fatty liver.

### Estimating Population Attributable Fraction

The population attributable fraction of obesity for a disease (i.e., the percent of patients having this disease due to obesity) depends on the relative risk of disease, and the prevalence of obesity. The following formula was used for the calculation:

$$\begin{array}{l} PAF=1-\left(\frac{1}{1-P+RR^{*}P}\;\right), \end{array}$$

where PAF stands for population attributable fraction, P for the prevalence of obesity, and RR for relative risk.

The proportion of population diseased attributable to obesity was calculated by multiplying the obesity attributable fraction (OAF) with the prevalence of the disease in the adult population for each sex.

(e.g., 61.8% of diabetic male patients are diabetic due to obesity (OAF), 13.86% of males have diabetes (prevalence), the calculation would be 61.8%^*^13.86% = *8.56% of the total population have diabetes due to obesity)*.

To calculate the number of patients who have the disease due to obesity, the proportion of population diseased attributable to obesity (PAF) was multiplied by the number of adult populations by sex. *(e.g., 8.56%* ^*^30 million = *2.75 million patients)*.

### Prevalence

Egypt National STEPwise Survey 2017 was utilized to obtain the prevalence of diabetes, hypertension and hyperlipidemia (10), which provided an estimate for the prevalence of total cholesterol ≥ 5.0 mmol/L, that was used as a proxy for hyperlipidemia. As the survey was based on measurements and not on interview, the total prevalence of these comorbidities could be estimated not only the diagnosed cases. For diabetes mellitus, the value was further adjusted to the proportion of type 2 only (11).

The global burden of disease (GBD) study prevalence estimates were used for most of the remaining diseases (11). These values were estimated for Egypt in 2017 and published by the Institute for Health Metrics and Evaluation (IHME) (12). Sleep apnea prevalence was estimated through a study by Franklin et al. in 2014 (13). Prevalence of fatty liver in Egypt was not found in the literature. We used a rough estimate of worldwide prevalence reported by Binobaid et al. in 2018 being 20–30% (14). We assumed Egypt would be in the midline and used the 25% value as an estimate for fatty liver prevalence in Egypt. Prevalence estimates and their sources are presented in Table 1.

**Table 1:** Prevalence of obesity and its comorbidities in Egypt.

**Disease**	**Prevalence total**	**Prevalence males**	**Prevalence females**	**References**
Obesity (BMI ≥30)	39.82%	29.51%	49.49%	100 million health survey 2019^a^
Diabetes mellitus	15.16%	13.86%	16.46%	Egypt National STEPwise Survey (10), GBD 2015 (15)
Hypertension	29.50%	29.80%	29.20%	Egypt National STEPwise Survey (10)
Ischemic heart disease (CHD/CAD)	2.71%	3.23%	2.16%	GBD 2015 (15)
Hyperlipidemia (total cholesterol ≥ 5.0 mmol/L)	19.20%	14.90%	23.40%	Egypt National STEPwise Survey (10)
stroke	1.74%	1.64%	1.86%	GBD 2015 (15)
Obstructive sleep apnea	NA	22.0%	17.0%	Franklin et al. (13)
Depression	4.16%	3.23%	5.15%	GBD 2015 (15)
Colon and colorectal cancer	0.030%	0.03%	0.03%	GBD 2015 (15)
Breast cancer	0.142%	0.00%	0.29%	GBD 2015 (15)
Endometrial cancer	0.016%	0.00%	0.03%	GBD 2015 (15)
Gall bladder disease	0.717%	0.46%	0.99%	GBD 2018 (15)
Osteoarthritis	5.022%	4.85%	5.20%	GBD 2018 (15)
Fatty liver	20–30%	25%	25%	Binobaid et al. (14)

*NA, not available*.

a *http://www.emro.who.int/ar/egy/egypt-events/hepatitis-c-elimination.html.*

### Relative Risks

Relative risks of diseases for obesity vs. no obesity were found in the literature. Most values were based on a meta-analysis conducted in 2009 by Guh et al. (16) Relative risk for obstructive sleep apnea was estimated to be seven-fold. (17) For fatty liver, ischemic heart disease and hyperlipidemia, values were abstracted from Li et al., (18) Jee et al. (19), and Fu et al. (20), respectively. Table 2 shows the relative risks of obesity associated diseases.

**Table 2:** Relative risks of diseases in people with obesity.

**Disease**	**Relative risk in males**	**Relative risk in females**	**References**
Diabetes mellitus	6.47	12.41	Guh et al. (16)
Hypertension	1.84	2.42	Guh et al. (16)
Ischemic heart disease	4.37	4.37	Jee et al. (19)
Hyperlipidemia	1.76	1.76	Fu et al. (20)
Stroke	1.51	1.49	Guh et al. (16)
Sleep apnea	7.00	7.00	Carol (17)
Depression	1.31	1.67	Guh et al. (16)
Colon and colorectal cancer	1.95	1.66	Guh et al. (16)
Breast cancer	-	1.13	Guh et al. (16)
Endometrial cancer	-	3.22	Guh et al. (16)
Gall bladder disease	1.43	2.32	Guh et al. (16)
Osteoarthritis	4.2	1.96	Guh et al. (16)
Fatty liver	3.53	3.53	Li et al. (18)

### Humanistic Burden

To estimate the number of deaths attributable to obesity, the obesity attributable fraction of each disease was multiplied by the number of deaths due to the disease in the total population. The estimated number of deaths due to each disease is published in the IHME database (12). We calculated the population attributable fraction of mortality only for major causes of death.

The global burden of disease study had estimates of DALY values in Egypt for several diseases (11). These values were multiplied by obesity attributable fraction to estimate the value of DALYs attributable to obesity.

Hyperlipidemia DALYs attributable to high cholesterol in the Eastern Mediterranean region was shown in a figure in the global health risks study (21). For obstructive sleep apnea (OSA), DALYs were based on a study that estimated DALYs lost due to sleep disorders among which obstructive sleep apnea is a main cause (22). When it came to fatty liver, cirrhosis was used as the main cause for DALY loss (23). Twenty percent of those affected by non-alcoholic fatty liver disease (NAFLD) eventually develop non-alcoholic steatohepatitis (NASH) (24). Of those, 20% develop cirrhosis (25). DALYs per cirrhotic patient in the Eastern Mediterranean region was used as a proxy for Egypt.

### Economic Burden

We focused on direct healthcare costs attributable to obesity. Direct non-healthcare costs, and indirect costs were not included in our analysis. We estimated the cost per patient for each disease, and then by sex we multiplied this value with the number of patients suffering from the disease and cared for and with the PAF. Then, we summed up the two values (males and females). We adjusted for the inflation when needed.

The costs of eight comorbidities (stroke, ischemic heart disease, depression, obstructive sleep apnea, osteoarthritis, endometrial cancer, gall bladder disease and fatty liver) were not available in the literature, so those costs were estimated through interviews with experts in each field.

For each comorbidity, the questionnaires for expert interviews were prepared based on cost components from published data. The questionnaires can be found in Supplementary Tables 2–9. The cost components were aggregated from the Health Insurance Organization (HIO) services price list, and the medication prices were aggregated from the public medication prices in the Egyptian market. Public prices were used to estimate medication costs because about 60% of the health spending in Egypt are out of pocket payments (26).

To avoid adding the costs for patients who are not treated, the cost values were multiplied by the percentage of treated population for each disease.

### Adjusting Values

Most values abstracted from the literature were from 2017 (last reported), so they were adjusted to 2020, based on the adult population growth factor. 2017 population data was found at CAPMAS (27), while 2020 population was an estimate through CIA world factbook (28). An exchange rate of 15.85 was used to exchange USD to EGP throughout the study.

## Results

### Clinical Burden

#### Prevalence of Obesity

According to “100 million health” survey, which was conducted in Egypt in 2019 and screened 49.7 million adult Egyptians (≥18 years old), 39.8% of adult Egyptians suffered from obesity (BMI ≥ 30 kg/m^2^). Obesity was more prevalent in adult females than adult males (49.5% of Egyptian adult females suffered obesity compared to 29.5% for males).

#### Patients With Diseases Attributable to Obesity

Diabetes mellitus type 2 had the highest prevalence attributable to obesity in adult Egyptians. Eighty five percent of females and 62% of males with diabetes mellitus type 2 can be attributed to obesity, translating to 4,759,839 and 3,004,347 females and males, respectively. About 13 million adults suffered from sleep apnea and about 8.5 million suffered from fatty liver due to obesity. Hypertension caused by obesity affected about 6 million adults. The estimated numbers of adult males and females who developed certain comorbidities due to obesity in 2020 in Egypt are shown in Table 3.

**Table 3:** Estimated numbers of adult males and females who suffered from a comorbidity due to obesity in 2020 in Egypt.

**Disease**	**PAF in males**	**PAF in females**	**Numbers in adult male population (2020)**	**Numbers in adult female population (2020)**	**Total population (2020)**
Diabetes mellitus	61.8%	85.0%	3,004,347	4,759,839	7,764,186
Hypertension	19.9%	41.3%	2,077,436	4,101,365	6,178,802
Ischemic heart disease	49.9%	62.5%	565,470	459,645	1,025,115
Hyperlipidemia	18.3%	27.3%	957,917	2,176,623	3,134,540
Stroke	13.1%	19.5%	75,234	123,304	198,538
Obstructive sleep apnea	63.9%	74.8%	4,933,804	4,327,953	9,261,757
Depression	8.4%	24.9%	94,937	436,033	530,970
Colon and colorectal cancer	21.9%	24.6%	2,587	2,185	4,772
Breast cancer	0.00	6.0%	-	6,031	6,031
Endometrial cancer	0.00	52.4%	-	5,944	5,944
Gall bladder disease	11.3%	39.5%	18,337	132,525	150,862
Osteoarthritis	48.6%	32.2%	826,810	570,284	1,397,094
Fatty liver	42.7%	55.6%	3,750,248	4,730,231	8,480,479

*PAF, population attributable fraction*.

#### Mortality Attributable to Obesity

In 2020, the estimated total number of deaths due to obesity was around 113 thousand which represents about 19% of the total estimated number of deaths in 2020 (29). Ischemic heart disease had the greatest share among obesity comorbidities causing mortalities, as it is a severe and common disease. The breakdown of mortality data is shown in Table 4.

**Table 4:** Mortality attributable to obesity.

**Disease**	**Death cases attributable to obesity**		Percentage of total deaths per year^*^	**Male: female ratio**
Diabetes mellitus	Total	7,877	1.31%	0.76
	Males	3,407		
	Females	4,470		
Ischemic heart disease	Total	94,575	15.71%	1.65
	Males	58,888		
	Females	35,687		
Stroke	Total	8,846	1.47%	0.89
	Males	4,171		
	Females	4,675		
Colon and colorectal cancer	Total	733	0.12%	1.3
	Males	414		
	Females	319		
Breast cancer	Total	243	0.04%	NA
	Males	-		
	Females	243		
Endometrial cancer	Total	192	0.03%	
	Males	-		NA
	Females	192		
Gall bladder disease	Total	341	0.06%	0.45
	Males	106		
	Females	235		
Total annual deaths attributable to obesity	Total	112,807	18.73%	1.48
	Males	66,986		
	Females	45,821		

* *Percentages are of the estimated total annual deaths in Egypt in 2020 (602,152 deaths)*.

### Humanistic Burden

#### Disability Adjusted Life Years

Ischemic heart disease was responsible for most DALYs attributable to obesity. This exceeded 2 million DALYs, as ischemic heart disease is a major contributor to premature mortality. Diabetes mellitus type 2 came next at about 660 thousand DALYs, as it is quite prevalent and affects quality of life considerably. Obstructive sleep apnea followed, with around 290 thousand DALYs, then stroke (240 thousand DALYs) and hyperlipidemia (230 thousand DALYs). Other comorbidities had less contribution to DALYs. The total DALYs attributable to obesity in 2020 was estimated to be about 4 million. The breakdown of DALYs data is shown in Table 5.

**Table 5:** DALYs attributable to obesity.

**Disease**	**DALYs in males**	**DALYs in females**	**Total DALYs**
Ischemic heart disease	1,504,408	847,929	2,352,337
Diabetes mellitus type 2	284,946	373,117	658,063
Obstructive sleep apnea	153,393	134,557	287,949
Stroke	107,235	128,708	235,943
Hyperlipidemia	95,149	137,667	232,816
Hypertension	37,598	58,945	96,543
Depression	16,499	75,811	92,310
Osteoarthritis	26,284	17,939	44,223
Fatty liver	12,405	16,131	28,536
Colon and colorectal cancer	12,499	9,683	22,182
Gall bladder disease	3,039	7,517	10,557
Breast cancer	-	9,225	9,225
Endometrial cancer	-	6,765	6,765
Total DALYS	2,253,455	1,823,993	4,077,448

*DALYs, disability adjusted life years*.

### Economic Burden

#### Diabetes Mellitus Type 2

According to a study conducted in 2017 (30, 31), the estimated healthcare cost of diabetes mellitus in Egypt was 22.3 Bn EGP in 2015. This value was divided by the number of diabetes patients in 2015 to estimate the cost per patient. This cost was adjusted to the inflation rate based on the consumer price index (32, 33) to year 2019—which was the most recent—to be 4,445 EGP. Finally, the cost per patient was multiplied by the estimated number of patients who had diabetes due to obesity in 2020 to reach about 34.5 Bn EGP.

#### Hypertension

In 2013, a study assumed that medications represent around 80% of the cost of hypertension in Egypt (34). The total cost of hypertension drugs in Egypt was abstracted from the monthly drug consumption report by IQVIA from IMS drug consumption report (unpublished data), showing that around 5.5 Bn EGP are spent on hypertension drugs annually in 2019. These values were used to calculate the annual burden of hypertension attributable to obesity in Egypt. The value was not adjusted to the number of diagnosed patients, since all patients taking medications are already diagnosed. From this the estimated cost of hypertension due to obesity was 2.1 Bn EGP.

#### Hyperlipidemia

The cost of hyperlipidemia treatment in Egypt was estimated to be around 9,109 EGP per patient annually (35). To estimate the total annual cost in Egypt, the proportion of treated patients was required, since some patients have the disease but are not treated or even diagnosed. The percentage of uncontrolled patients was estimated through a US based study (65.2%) (36). This was used to estimate the total number treated in Egypt.

#### Colon and Colorectal Cancer

The estimated average cost of treating colorectal cancer per patient annually was 610 USD in Egypt (37). This was used to estimate the cost for patients attributable to obesity. The percentage of diagnosed cases was calculated based on an epidemiological study in Egypt to be around 15% (38).

#### Breast Cancer

The average total cost of treating breast cancer in Egypt was estimated at 46,910 USD per patient (39). However, the annual cost was not indicated in the report, so the total cost was divided by the average number of years lived by breast cancer patients (6.06 years). This value was calculated from the data available through Globocan 2018 (40), resulting in an annual cost of 7,741 USD per patient.

To find the percentage of diagnosed cases, the number of diagnosed cases (38) was divided by the prevalence.

Table 6 shows the annual direct healthcare cost of obesity comorbidities. Diabetes mellitus and hyperlipidemia are responsible for the largest financial burden among the comorbidities, representing about 60% of the total direct health care cost. Osteoarthritis is a major contributor also to the total economic burden of obesity. The total annual cost of treatment of diseases attributable to obesity was estimated to be 62,413 million EGP.

**Table 6:** Annual direct healthcare cost due to obesity.

**Comorbidity**	**Annual cost of treatment attributable to obesity/million EGP**	**Percentage of total attributable cost**	**Source of annual cost estimation per patient**
Diabetes mellitus	34,512	55.3%	Assaad et al. (30)
Hyperlipidemia	9,936	15.9%	Shaheen et al. (35)
Osteoarthritis	7,876	12.6%	Estimated based on expert interviews
Fatty liver	2,881	4.6%	Estimated based on expert interviews
Obstructive sleep apnea	2,650	4.2%	Estimated based on expert interviews
Hypertension	2,097	3.4%	Ibrahim (34)
Ischemic heart disease	1,624	2.6%	Estimated based on expert interviews
Depression	440	0.7%	Estimated based on expert interviews
Stroke	269	0.4%	Estimated based on expert interviews
Breast cancer	101	0.2%	Skrundevskiy et al. (39)
Gall bladder disease	18	0.0%	Estimated based on expert interviews
Colon and colorectal cancer	6	0.0%	Abotaleb (37)
Endometrial cancer	2	0.0%	Estimated based on expert interviews
Total	62,413 million EGP per year	100%	

## Discussion

Obesity is a leading cause of preventable death globally; (1) and Egypt is one of the leading countries in the prevalence of obesity worldwide (3). The burden of obesity might be overseen because obesity acts as a proxy to many comorbidities which in turn causes morbidity and mortality. We found no studies focused on evaluating the burden of obesity in Egypt. Our study focused on estimating the economic, and humanistic burden of obesity in Egypt. Estimating the magnitude of the issue is especially important to grab both the public attention, and decision makers to both facilitate individual actions and implement public health policies to alleviate the burden of obesity in Egypt.

The prevalence of obesity has increased in adults in Egypt to reach about 40% according to 100million health survey (2019) compared to the 36% estimate of 2017 STEPwise survey (10). Obesity is more prevalent in Egyptian females compared to males (about 50% compared to 30% respectively). This could be probably attributed to the cultural factors, as females in Egypt are less likely to be involved in physical activities compared to males (10, 41).

The results revealed that nearly three quarters of adult diabetes mellitus type 2 cases in Egypt are attributable to obesity. Obesity is also the underlying disease for more than half of obstructive sleep apnea, ischemic heart disease, endometrial cancer and fatty liver cases in Egypt.

The comorbidities of obesity carry a substantial mortality risk causing around 19% of annual mortalities in Egypt. Although female prevalence is higher, 59% of obesity attributable deaths are males. The main reason for that is ischemic heart disease, which caused around 84% of deaths attributable to obesity, and is dominant in males, with a male to female ratio of 1.65.

Ischemic heart disease also contributes to the major portion of DALYs lost because of the early mortality, followed by diabetes mellitus which makes about 16% of total DALYs. Although obstructive sleep apnea does not contribute to death, it makes up about 7% of total DALYs.

Concerning the economic burden of obesity, chronic diseases have the lion's share, with diabetes mellitus type 2 accounting for more than half of the annual cost followed by hyperlipidemia, and osteoarthritis at around 30% combined. Although cancer treatment is comparatively more expensive, cancers did not make up a large chunk in either costs, DALYs, or deaths because of their lower prevalence compared to chronic diseases.

Our study shows that there has been an increase in the burden of obesity in recent years, where the burden of obesity in the Eastern Mediterranean region study in 2015 (15) estimated lower values for high BMI attributable deaths and DALYs than the our study. In 2015, the study estimated 90,774 attributable deaths in Egypt, while our results showed an increase to reach 112,807 attributable deaths in 2020, translating to an increase from 16 (42) to 19% of the total annual deaths. For DALYs, an increase was also observed reaching about 1 million more DALYs lost. After adjusting to population growth, obesity attributable DALYs increased from 3.29 to 3.92% in the total population. Our methodology is a bit different than the above-mentioned study, but the results are consistent and show that obesity causes tremendous losses concerning mortality and quality of lives.

Our findings have some policy implications, as they show that effective prevention of obesity in Egypt would result in a huge health benefit. There are several policies and recommendations that could be applied to decrease the prevalence of obesity. The following examples from a recent systematic review could be implemented in Egypt: incentivizing healthy food, portion size restrictions, taxation and dedicated tax revenue for obesity prevention, promoting physical activity, certain food and beverage restriction combined with nutrition education at schools (43). The WHO regional office has published proposed policy priorities for preventing obesity and diabetes in the Eastern Mediterranean Region in 2017 (44). The policy recommendations included in this report could help in decreasing obesity and its burden if implemented. Learnings and recommendations about health policies and interventions from other countries aiming for obesity reduction can be beneficial; however, first, the feasibility of selected policies or health interventions needs to be evaluated nationally from both a regulatory and cultural perspective.

Our study has certain limitations. Due to the lack of complete and reliable local data, it was based on a mixture of primary and secondary data. As many less important comorbidities were not included in the analysis, our disease burden estimates are conservative from this perspective. Since some comorbidities mainly lead to death via other diseases and complications (e.g., hypertension, hyperlipidemia, sleep apnea, depression, osteoarthritis, and fatty liver), the number of deaths directly coded as death due to these diseases underestimate their true role in the mortality statistics. This way we somewhat underestimated the true population fraction of mortality, but only to a small extent, as the major consequences of these conditions were included as comorbidities in the calculations. On the other hand, we could not avoid double counting some cost items, as the cost of diabetes included the cost of the management of its complications, too. Unfortunately, the cost of complications of diabetes was not available by complication, thus we could not correct for this. However, as only a fraction of strokes and ischemic heart diseases is due to diabetes and the proportion of the cost of these diseases is only small in the total direct healthcare cost of obesity, the size of the error is minor. Also, direct non-healthcare costs due to obesity, as well as indirect costs were not considered in this study, this way counterbalancing the double counting of some other cost items. A limited number of expert interviews were conducted to estimate the annual cost of comorbidities, which limits the generalizability of the cost data. Further questionnaires and research should be conducted to have better estimates of comorbidities' annual cost.

Some methodological issues contribute to limitations, too. The adult population definition was different among different sources; 100 million health survey included adults aged not <18, while STEPwise survey included adults aged 15–69 and sleep apnea was estimated for a population aged 30–60 years old. Also, hyperlipidemia DALY values were found for a population above 30 only and were estimated for hypercholesterolemia not hyperlipidemia which were assumed equal. For the relative risks of obstructive sleep apnea and fatty liver, there was no differentiation by sex. For fatty liver disease, the references did not mention the breakdown of prevalence by males and females. In these cases, it was assumed that the values are equal for both sexes. The DALY calculation of fatty liver disease was based on the burden caused by liver cirrhosis. Some estimates were not found specifically for Egypt, then values from other countries were used. For hyperlipidemia, the exact percentage of untreated patients was not available, so a proxy was used, based on the percentage of uncontrolled population. Nevertheless, these minor technical issues could not substantially bias the results. Furthermore, we believe, that utilization of these data for research facilitates the improvement of the quality of data.

In conclusion, obesity is a major contributor to ill-health and health expenditure in Egypt. It is a risk factor of several diseases, of which some are very severe, like heart diseases and stroke, and other are milder but highly prevalent among people with obesity. Consequently, obesity causes huge clinical, humanistic, and economic burden to Egyptians as individuals and as a society. More studies are required to evaluate different interventions and policies that can reduce obesity in Egypt and alleviate the impact of obesity on health.

## Data Availability Statement

Publicly available datasets were analyzed in this study. This data can be found at: Several data sources were used in the article. Some came from publicly available datasets found in the references. Others came from a third party. The data that came from a third party are: 1-Data from 100 M health survey, Requests to access these datasets should be directed to [Galal Elshishiney, elshishiney@yahoo.com] 2-Data from IMS reports through Novo Nordisk, Requests to access these datasets should be directed to [Waleed Taha, wtah@novonordisk.com].

## Ethics Statement

Ethical review and approval was not required for the study on human participants in accordance with the local legislation and institutional requirements. Written informed consent for participation was not required for this study in accordance with the national legislation and the institutional requirements.

## Author Contributions

ZV, SA, AF, BE, and EB conceptualized the project protocol. AF and BE conducted the analysis and calculations. MA, AE, IE, NE, and EA-M were the experts consulted throughout the steps of research process. GE advised for the data coming from national surveys. BE prepared the draft manuscript. ZV supervised all the scientific steps and provided comments. DT ensured the medical accuracy of the information. All authors revised, commented and approved the final manuscript.

## Conflict of Interest

Syreon Middle East was a contractual partner of Novo Nordisk Egypt. AF, SA, and ZV are shareholders in Syreon Middle East. BE is an employee at Syreon Middle East. DT and EB are employees at Novo Nordisk Egypt. The remaining authors declare that the research was conducted in the absence of any commercial or financial relationships that could be construed as a potential conflict of interest.

## Publisher's Note

All claims expressed in this article are solely those of the authors and do not necessarily represent those of their affiliated organizations, or those of the publisher, the editors and the reviewers. Any product that may be evaluated in this article, or claim that may be made by its manufacturer, is not guaranteed or endorsed by the publisher.
