# Supplementary material for: The Burden of Obesity in Egypt
Source: Front Public Health. 2021 Aug 27;9:718978. doi: 10.3389/fpubh.2021.718978 (PMC8429929; doi:10.3389/fpubh.2021.718978)
Supplement: Supplementary file 1 [file Data_Sheet_1.ZIP › Table S1 search strategy.docx]

Table S1 Search key for obesity comorbidities

| Domains | **obesity** | **comorbidities** |
| --- | --- | --- |
| synonyms | obesity OR overweight OR high BMI or BMI >30 or BMI ≥ 30 | comorbidity OR "related diseases" OR comorbidities OR "attributable disease" OR "associated diseases" |
| Search terms | | |
| Google | (obesity OR overweight OR high BMI or BMI >30 or BMI ≥ 30) AND (comorbidity OR "related diseases" OR comorbidities OR "attributable disease" OR "associated diseases") | |
| Google scholar | (obesity OR overweight OR high BMI or BMI >30 or BMI ≥ 30) AND (comorbidity OR "related diseases" OR comorbidities OR "attributable disease" OR "associated diseases") | |
| PubMed | (obesity[Title/Abstract] OR overweight[Title/Abstract] OR high BMI[Title/Abstract] OR BMI >30[Title/Abstract] OR BMI ≥ 30)[Title/Abstract] AND (comorbidity[Title/Abstract] OR "related diseases"[Title/Abstract] OR comorbidities[Title/Abstract] OR "attributable disease"[Title/Abstract] OR "associated diseases")[Title/Abstract] | |
| BMI: Body Mass Index | | |
