# Supplementary material for: The Burden of Obesity in Egypt
Source: Front Public Health. 2021 Aug 27;9:718978. doi: 10.3389/fpubh.2021.718978 (PMC8429929; doi:10.3389/fpubh.2021.718978)
Supplement: Supplementary file 1 [file Data_Sheet_1.ZIP › Table S2 IHD cost questionnaire.docx]

Table S2 Questionnaire for medical cost of ischemic heart disease patient per year

| **Cost** **Element** | **Unit cost** | **Percentage of patients who utilize it** | **Frequency/ year** | **Total** **Cost/ year (calculation)** |  |
| --- | --- | --- | --- | --- | --- |
| Echocardiogram |  |  |  |  |  |
| Stress echocardiogram |  |  |  |  |  |
| Electrocardiogram |  |  |  |  |  |
| Exercise test |  |  |  |  |  |
| Holter |  |  |  |  |  |
| Ambulatory Blood Pressure Monitoring |  |  |  |  |  |
| Venous color Doppler ultrasound |  |  |  |  |  |
| **Laboratory tests** |  |  |  |  |  |
| CK |  |  |  |  |  |
| CK-MB |  |  |  |  |  |
| HDL and LDL cholesterol |  |  |  |  |  |
| Total cholesterol |  |  |  |  |  |
| Creatinine |  |  |  |  |  |
| Triglycerides |  |  |  |  |  |
| Troponin |  |  |  |  |  |
| **Medications** |  |  |  |  |  |
| ***Antiplatelets*** |  |  |  |  |  |
| ASA |  |  |  |  |  |
| Clopidogrel 75 mg. |  |  |  |  |  |
| Ticagrelor 90 mg |  |  |  |  |  |
| Ticagrelor 60 mg |  |  |  |  |  |
| ***Hydro pyridines*** ***CCBs*** |  |  |  |  |  |
| Amlodipine |  |  |  |  |  |
| ***Non-Dihydropyridine CCBs*** |  |  |  |  |  |
| Verapamil 80 mg |  |  |  |  |  |
| ***Diuretics*** |  |  |  |  |  |
| Furosemide 40 mg |  |  |  |  |  |
| Other (Torsemide 10 mg) |  |  |  |  |  |
| ***Beta blockers*** |  |  |  |  |  |
| nebivolol 5 mg. |  |  |  |  |  |
| nebivolol 10 mg. |  |  |  |  |  |
| Metoprolol 100 mg. |  |  |  |  |  |
| Carvedilol 6.25 mg |  |  |  |  |  |
| Carvedilol 25 mg |  |  |  |  |  |
| ***ACE inhibitors*** |  |  |  |  |  |
| Enalapril 20 mg |  |  |  |  |  |
| other (Perindopril 5 mg) |  |  |  |  |  |
| ***ARBs*** |  |  |  |  |  |
| valsartan 40 mg. |  |  |  |  |  |
| Candesartan 4 mg. |  |  |  |  |  |
| Candesartan 8 mg. |  |  |  |  |  |
| others (Candesartan 16 mg+ hydrochlorothiazide) |  |  |  |  |  |
|  |  |  |  |  |  |
| Nicorandil 10 mg. (nitrates like action) |  |  |  |  |  |
| ***Statins*** |  |  |  |  |  |
| Atorvastatin 40mg |  |  |  |  |  |
| Rosuvastatin 20 mg |  |  |  |  |  |
| ***Anticoagulants*** |  |  |  |  |  |
| Warfarin 3mg |  |  |  |  |  |
| **NOAC** |  |  |  |  |  |
| dabigatran 150 mg |  |  |  |  |  |
| rivaroxaban 20 mg |  |  |  |  |  |
| apixaban 5 mg |  |  |  |  |  |
| ***Antianginal*** |  |  |  |  |  |
| trimetazidine |  |  |  |  |  |
| Ivabradine 5 mg |  |  |  |  |  |
|  |  |  |  |  |  |
| pantoprazole |  |  |  |  |  |
| Paracetamol |  |  |  |  |  |
| Amiodarone |  |  |  |  |  |
| **Hospitalization days** |  |  |  |  |  |
| **Emergency room visits** |  |  |  |  |  |
| **Procedures** |  |  |  |  |  |
| CABG |  |  |  |  |  |
| PCI and angioplasty/ stenting |  |  |  |  |  |
| **Outpatient care** |  |  |  |  |  |
| Consultant (cardiologist) |  |  |  |  |  |
| cardiothoracic surgeon |  |  |  |  |  |
| Total cost/year | | | |  | |
