# Supplementary material for: The Burden of Obesity in Egypt
Source: Front Public Health. 2021 Aug 27;9:718978. doi: 10.3389/fpubh.2021.718978 (PMC8429929; doi:10.3389/fpubh.2021.718978)
Supplement: Supplementary file 1 [file Data_Sheet_1.ZIP › Table S3 depression cost questionnaire.docx]

Table S3 Questionnaire for medical cost of depression patient per year

| cost-element | **Unit cost** | **Mild-moderate** | | **Severe** | | **Suicidal Ideation (SI)** |  | **Total Cost/year** |
| --- | --- | --- | --- | --- | --- | --- | --- | --- |
|  |  | Percentage | Freq. | Percentage | Freq. | Percentage | Freq. |  |
|  |  |  |  |  |  |  |  |  |
| Psychotherapist |  |  |  |  |  |  |  |  |
| Antipsychotics |  |  |  |  |  |  |  |  |
| amitriptyline 25 mg |  |  |  |  |  |  |  |  |
| clomipramine 25 mg |  |  |  |  |  |  |  |  |
| clomipramine 75 mg |  |  |  |  |  |  |  |  |
| fluvoxamine 50 mg. |  |  |  |  |  |  |  |  |
| citalopram 20 mg. |  |  |  |  |  |  |  |  |
| escitalopram 10mg |  |  |  |  |  |  |  |  |
| Duloxetine 60 mg |  |  |  |  |  |  |  |  |
| venlafaxine 150 mg XR |  |  |  |  |  |  |  |  |
| Mirtazapine 30 mg. |  |  |  |  |  |  |  |  |
| Mianserin 30 mg. |  |  |  |  |  |  |  |  |
| trazodone 50 mg |  |  |  |  |  |  |  |  |
| trazodone 100 mg |  |  |  |  |  |  |  |  |
| Clozapine25mg.qd |  |  |  |  |  |  |  |  |
| Clozapine 100 mg. |  |  |  |  |  |  |  |  |
| olanzapine 10mg.qd |  |  |  |  |  |  |  |  |
| quetiapine 100 mg. |  |  |  |  |  |  |  |  |
| alprazolam0.25 mg. |  |  |  |  |  |  |  |  |
| alprazolam 0.5 mg. |  |  |  |  |  |  |  |  |
| Bromazepam 1.5mg |  |  |  |  |  |  |  |  |
| Bromazepam 3mg |  |  |  |  |  |  |  |  |
| Muscle relaxants (e.g. Myofen) |  |  |  |  |  |  |  |  |
| Analgesics (paracetamol) |  |  |  |  |  |  |  |  |
| For tremors (e.g. Achtenon 2 mg.) |  |  |  |  |  |  |  |  |
| hospitalization (Days) |  |  |  |  |  |  |  |  |
| **Treatment for hospitalized patients** |  |  |  |  |  |  |  |  |
| clozapine 25 mg |  |  |  |  |  |  |  |  |
| clozapine 100mg |  |  |  |  |  |  |  |  |
| long acting haloperidol, I.M injection |  |  |  |  |  |  |  |  |
| Risperidone 3 mg |  |  |  |  |  |  |  |  |
| Risperidone injection |  |  |  |  |  |  |  |  |
| For tremors (e.g. Achtenon 2 mg) |  |  |  |  |  |  |  |  |
| Total cost/year | | | | | | | |  |
