# Supplementary material for: The Burden of Obesity in Egypt
Source: Front Public Health. 2021 Aug 27;9:718978. doi: 10.3389/fpubh.2021.718978 (PMC8429929; doi:10.3389/fpubh.2021.718978)
Supplement: Supplementary file 1 [file Data_Sheet_1.ZIP › Table S4 osteoarthritis cost questionnaire.docx]

Table S4 Questionnaire for medical cost of osteoarthritis per patient per year

| **Data** |  | **frequency** | | | | | | | | | |  |  |  |
| --- | --- | --- | --- | --- | --- | --- | --- | --- | --- | --- | --- | --- | --- | --- |
| **Direct Costs** |  | **Kellgren-Lawrence criteria** | | | | | | | |  |  |  |  |  |
|  | **Cost** | **Grade 1** | | **Grade 2** | | **Grade 3** | | **Grade 4** | | **Prosthetic** | | **Repeated annual cost** | **One time cost** | **Total Average Cost** |
| **% of pts. In each grade** |  |  | |  | |  | |  | |  | |  |  |  |
|  |  | **%** | **freq./ year** | **%** | **freq./year** | **%** | **freq./ year** | **%** | **freq./ year** | **%** | **freq./ year** |  |  |  |
| **Diagnostics** |  |  |  |  |  |  |  |  |  |  |  |  |  |  |
| ***Biological*** ***testing*** |  |  |  |  |  |  |  |  |  |  |  |  |  |  |
| Antinuclear antibody (ANA) |  |  |  |  |  |  |  |  |  |  |  |  |  |  |
| Anti-cyclic citrullinated peptide (anti-CCP) |  |  |  |  |  |  |  |  |  |  |  |  |  |  |
| Uric acid |  |  |  |  |  |  |  |  |  |  |  |  |  |  |
| Erythrocyte sedimentation rate (ESR) |  |  |  |  |  |  |  |  |  |  |  |  |  |  |
| C-reactive protein (CRP) |  |  |  |  |  |  |  |  |  |  |  |  |  |  |
| Lyme serology |  |  |  |  |  |  |  |  |  |  |  |  |  |  |
| ***Imaging*** |  |  |  |  |  |  |  |  |  |  |  |  |  |  |
| Radiographs (X ray) |  |  |  |  |  |  |  |  |  |  |  |  |  |  |
| MRI |  |  |  |  |  |  |  |  |  |  |  |  |  |  |
| other(ultrasound) |  |  |  |  |  |  |  |  |  |  |  |  |  |  |
| **Surgery** |  |  |  |  |  |  |  |  |  |  |  |  |  |  |
| total Joint Revision |  |  |  |  |  |  |  |  |  |  |  |  |  |  |
| Hospitalization length of stay (days) |  |  |  |  |  |  |  |  |  |  |  |  |  |  |
| **Outpatient visits** |  |  |  |  |  |  |  |  |  |  |  |  |  |  |
| GP (in HIO, to give him a new prescription only) |  |  |  |  |  |  |  |  |  |  |  |  |  |  |
| Rheumatologists |  |  |  |  |  |  |  |  |  |  |  |  |  |  |
| Orthopedists |  |  |  |  |  |  |  |  |  |  |  |  |  |  |
| ER visits |  |  |  |  |  |  |  |  |  |  |  |  |  |  |
| **Pharmacological TTT** |  |  |  |  |  |  |  |  |  |  |  |  |  |  |
| *Oral agents* | ***cost/ 1 tab.*** | **% of pts**. | **Annual no of tablets** | **% of pts**. | **Annual no of tablets** | **% of pts**. | **Annual no of tablets** | **% of pts**. | **Annual no of tablets** | **% of pts**. | **Annual no of tablets** |  |  |  |
| Acetaminophen |  |  |  |  |  |  |  |  |  |  |  |  |  |  |
| celecoxib 200 mg. qd |  |  |  |  |  |  |  |  |  |  |  |  |  |  |
| Diclofenac Sodium 50mg. Bid |  |  |  |  |  |  |  |  |  |  |  |  |  |  |
| meloxicam 15 mg. |  |  |  |  |  |  |  |  |  |  |  |  |  |  |
| Antiepileptic drugs |  |  |  |  |  |  |  |  |  |  |  |  |  |  |
| Gabapentin 100 mg |  |  |  |  |  |  |  |  |  |  |  |  |  |  |
| Gabapentin 400 mg |  |  |  |  |  |  |  |  |  |  |  |  |  |  |
| Selected antidepressants (duloxetine 60 mg.) |  |  |  |  |  |  |  |  |  |  |  |  |  |  |
| Corticosteroids (Disprelone 15 mg.) |  |  |  |  |  |  |  |  |  |  |  |  |  |  |
| Glucosamine/chondroitin |  |  |  |  |  |  |  |  |  |  |  |  |  |  |
| *Injectable agents* |  |  |  |  |  |  |  |  |  |  |  |  |  |  |
| Corticosteroids |  |  |  |  |  |  |  |  |  |  |  |  |  |  |
| Hyaluronic acid (Visco supplementation) |  |  |  |  |  |  |  |  |  |  |  |  |  |  |
| Administration of hyaluronic acid injection |  |  |  |  |  |  |  |  |  |  |  |  |  |  |
| *Topical agents* |  |  |  |  |  |  |  |  |  |  |  |  |  |  |
| Corticosteroids |  |  |  |  |  |  |  |  |  |  |  |  |  |  |
| Methyl salicylate |  |  |  |  |  |  |  |  |  |  |  |  |  |  |
| Diclofenac |  |  |  |  |  |  |  |  |  |  |  |  |  |  |
| Glucosamine/chondroitin |  |  |  |  |  |  |  |  |  |  |  |  |  |  |
| non-pharmacological TTT. (physiotherapy) |  |  |  |  |  |  |  |  |  |  |  |  |  |  |
| Costs of side effects from treatments |  |  |  |  |  |  |  |  |  |  |  |  |  |  |
| gastric discomfort (PPIs) |  |  |  |  |  |  |  |  |  |  |  |  |  |  |
| gastric ulcer + prophylaxis (PPIs) |  |  |  |  |  |  |  |  |  |  |  |  |  |  |
| Technical aids |  |  |  |  |  |  |  |  |  |  |  |  |  |  |
| Knee braces |  |  |  |  |  |  |  |  |  |  |  |  |  |  |
| Orthopedic sole |  |  |  |  |  |  |  |  |  |  |  |  |  |  |
| cane |  |  |  |  |  |  |  |  |  |  |  |  |  |  |
| wheelchair |  |  |  |  |  |  |  |  |  |  |  |  |  |  |
| Total cost/ year | | | | | | | | | | | | | |  |
