# Supplementary material for: The Burden of Obesity in Egypt
Source: Front Public Health. 2021 Aug 27;9:718978. doi: 10.3389/fpubh.2021.718978 (PMC8429929; doi:10.3389/fpubh.2021.718978)
Supplement: Supplementary file 1 [file Data_Sheet_1.ZIP › Table S5 endometrial cancer cost questionnaire.docx]

Table S5 Questionnaire for medical cost of endometrial cancer per patient per year

|  | Unit cost | Weight | Frequency | Final cost |
| --- | --- | --- | --- | --- |
| **Outpatient visit** |  |  |  |  |
| GP |  |  |  |  |
| Gynecologist visit |  |  |  |  |
| Oncologist visit |  |  |  |  |
| Hospitalization |  |  |  |  |
| **Lab tests** |  |  |  |  |
| a) Prothrombin time |  |  |  |  |
| b) Partial thromboplastin |  |  |  |  |
| c)Pregnancy testing |  |  |  |  |
| CBC |  |  |  |  |
| Liver/Renal function |  |  |  |  |
| Viruses C and B |  |  |  |  |
| Random blood sugar |  |  |  |  |
| **Diagnostic studies** |  |  |  |  |
| a) Transvaginal Ultrasonography |  |  |  |  |
| b) Endometrial Biopsy |  |  |  |  |
| c)Hysteroscopy |  |  |  |  |
| d)MRI |  |  |  |  |
|  |  |  |  |  |
| **Treatment** |  |  |  |  |
| a) Surgery |  |  |  |  |
| b) Laparoscopic surgery |  |  |  |  |
| c)Adjuvant chemotherapy |  |  |  |  |
| Doxorubicin |  |  |  |  |
| Cisplatin |  |  |  |  |
| Docetaxel |  |  |  |  |
| Cyclophosphamide |  |  |  |  |
| Ifosfamide |  |  |  |  |
| Nedaplatin |  |  |  |  |
| Irinotecan |  |  |  |  |
| Other drugs |  |  |  |  |
| d) hormonal therapy |  |  |  |  |
| e) Adjuvant Radiotherapy |  |  |  |  |
| Total cost |  | | | |
