# Supplementary material for: The Burden of Obesity in Egypt
Source: Front Public Health. 2021 Aug 27;9:718978. doi: 10.3389/fpubh.2021.718978 (PMC8429929; doi:10.3389/fpubh.2021.718978)
Supplement: Supplementary file 1 [file Data_Sheet_1.ZIP › Table S6 obstructive sleep apnea questionnaire.docx]

Table S6 Questionnaire for medical cost of obstructive sleep apnea per patient per year

|  | Unit cost | Weight | Frequency | Final cost |
| --- | --- | --- | --- | --- |
| **Outpatient visit** |  |  |  |  |
| GP visit |  |  |  |  |
| Specialist visit |  |  |  |  |
| Hospitalization |  |  |  |  |
| **Diagnostic procedures** |  |  |  |  |
| Overnight sleep study |  |  |  |  |
| **Medical device related costs** |  |  |  |  |
| Purchase of CPAP |  |  |  |  |
| **CPAP accessories** |  |  |  |  |
| a) Headgear and mask |  |  |  |  |
| b) Tubing |  |  |  |  |
| c)Heated humidifier |  |  |  |  |
| d)minor attendances by technician |  |  |  |  |
| **Pharmaceutical treatment if any** |  |  |  |  |
| **Drug 1 ……………..** |  |  |  |  |
| **Drug 2 ………………** |  |  |  |  |
| Total cost |  | | | |
