# Supplementary material for: The Burden of Obesity in Egypt
Source: Front Public Health. 2021 Aug 27;9:718978. doi: 10.3389/fpubh.2021.718978 (PMC8429929; doi:10.3389/fpubh.2021.718978)
Supplement: Supplementary file 1 [file Data_Sheet_1.ZIP › Table S7 stroke cost questionnaire.docx]

Table S7 Questionnaire for medical cost of stroke per patient per year*

| Data |  |  |  |  |  |
| --- | --- | --- | --- | --- | --- |
| **Diagnostics** | **Unit Cost** |  | % of pts | Frequency | Total cost |
| ***Blood Tests*** |  |  |  |  |  |
| CBC |  |  |  |  |  |
| PT |  |  |  |  |  |
| PTT |  |  |  |  |  |
| INR |  |  |  |  |  |
| glucose |  |  |  |  |  |
| Sodium |  |  |  |  |  |
| Potassium |  |  |  |  |  |
| Calcium |  |  |  |  |  |
| LFT |  |  |  |  |  |
| sr.cr |  |  |  |  |  |
| BUN |  |  |  |  |  |
| ***Imaging*** |  |  |  |  |  |
| Cranial CT |  |  |  |  |  |
| Cranial MRI |  |  |  |  |  |
| Extracranial Doppler sonography |  |  |  |  |  |
| Echocardiography |  |  |  |  |  |
| **Rehospitalization (recurrent stroke)** |  |  |  |  |  |
| ICU day cost |  |  |  |  |  |
| Stroke or intermediate care unit /day |  |  |  |  |  |
| General ward daily cost |  |  |  |  |  |
| **Medication** |  |  |  |  |  |
| Alteplase (e.g Activase, Actilyse) |  |  |  |  |  |
| Ambulance |  |  |  |  |  |
| **Procedures** |  |  |  |  |  |
| catheter-delivered (tPA) to the site of plaque |  |  |  |  |  |
| Aggregated Hospital Cost |  |  |  |  |  |
| **outpatient (at 3months)** |  |  |  |  |  |
| **Outpatient rehabilitation** |  |  |  |  |  |
| Nursing |  |  |  |  |  |
| Physiotherapy |  |  |  |  |  |
| Speech therapy |  |  |  |  |  |
| Specialist care |  |  |  |  |  |
| **Medications** |  |  |  |  |  |
| Warfarin(3mg) |  |  |  |  |  |
| Aspirin (100mgqd) |  |  |  |  |  |
| Clopidogrel (75mgqdfor1yr.) |  |  |  |  |  |
| atorvastatin (20mg&40mgqd) |  |  |  |  |  |
| rosuvastatin (20mg.qd) |  |  |  |  |  |
| simvastatin (20mgqd) |  |  |  |  |  |
| ACEi (enalapril20mg.qd, Ramipril5mg.qd) |  |  |  |  |  |
| ARBs (Angiotensin receptor blockers) |  |  |  |  |  |
| Betablockers (Bisoprolol5mgqd) |  |  |  |  |  |
| CCB (Amlodipine5mgQD) |  |  |  |  |  |
| **Appliances** |  |  |  |  |  |
| wheelchair |  |  |  |  |  |
| walking aid (Cane) |  |  |  |  |  |
| shoulder support |  |  |  |  |  |
| Aggregated outpatient Cost (3 months) |  |  |  |  |  |
| **outpatient (at 12 months)** |  |  |  |  |  |
| **Medications** |  |  |  |  |  |
| Warfarin (3mg) |  |  |  |  |  |
| Dabigatran (150 mg bds) |  |  |  |  |  |
| Rivaroxaban(20mgqd) |  |  |  |  |  |
| Apixaban(5mg.bds) |  |  |  |  |  |
| ASA(100mgqd) |  |  |  |  |  |
| atorvastatin (20mg.40mg.qd) |  |  |  |  |  |
| rosuvastatin (20mg.qd) |  |  |  |  |  |
| simvastatin (20mg.qd) |  |  |  |  |  |
| Enalapril20mg.qd |  |  |  |  |  |
| Ramipril5mg.qd |  |  |  |  |  |
| ARBs |  |  |  |  |  |
| Betablockers (Bisoprolol5mgqd) |  |  |  |  |  |
| CCB(Amlodipine5mg.) |  |  |  |  |  |
| Aggregated outpatient Cost (12 months) |  |  |  |  |  |
| Total cost of **stroke** |  |  |  |  |  |

*If the values are provided by physicians as per stroke event, the value was multiplied by the probability of stroke incidence per year to find the average annual cost.
