# Supplementary material for: The Burden of Obesity in Egypt
Source: Front Public Health. 2021 Aug 27;9:718978. doi: 10.3389/fpubh.2021.718978 (PMC8429929; doi:10.3389/fpubh.2021.718978)
Supplement: Supplementary file 1 [file Data_Sheet_1.ZIP › Table S8 fatty liver cost questionnaire.docx]

Table S8 Questionnaire for medical cost of fatty liver disease per patient per year

|  | Unit cost | Weight | Frequency | Final cost |
| --- | --- | --- | --- | --- |
| **Visits** |  |  |  |  |
| GP |  |  |  |  |
| Specialist visit |  |  |  |  |
| Hospitalization |  |  |  |  |
| Follow up |  |  |  |  |
| **Lab tests** |  |  |  |  |
| Alanine transaminase (ALT) |  |  |  |  |
| Aspartate aminotransferase (AST) |  |  |  |  |
| Controlled attenuation parameter |  |  |  |  |
| Fatty liver index |  |  |  |  |
| Gamma-glutamyl transferase (GGT) |  |  |  |  |
| Steato Test |  |  |  |  |
| NAFLD liver fat score |  |  |  |  |
| Fibrosis biomarkers |  |  |  |  |
| CBC |  |  |  |  |
| Fasting and random blood glucose |  |  |  |  |
| Viruses B and C |  |  |  |  |
| PT |  |  |  |  |
| PTT |  |  |  |  |
| INR |  |  |  |  |
| Urea |  |  |  |  |
| Creatinine |  |  |  |  |
| Imaging |  |  |  |  |
| Ultrasound |  |  |  |  |
| MRI |  |  |  |  |
| MRS (Magnetic resonance spectroscopy) |  |  |  |  |
| triphasic CT scan |  |  |  |  |
| Procedures |  |  |  |  |
| Liver biopsy |  |  |  |  |
| Management |  |  |  |  |
| Dietary changes |  |  |  |  |
| **Pharmacotherapy** |  |  |  |  |
| Bioglutathone |  |  |  |  |
| Vit E 400 & silymarin 140 mg |  |  |  |  |
| Others |  |  |  |  |
| **Surgery** |  |  |  |  |
| Bariatric surgery |  |  |  |  |
| **exercise and control** |  |  |  |  |
| Total cost |  | | | |
