# Supplementary material for: The Burden of Obesity in Egypt
Source: Front Public Health. 2021 Aug 27;9:718978. doi: 10.3389/fpubh.2021.718978 (PMC8429929; doi:10.3389/fpubh.2021.718978)
Supplement: Supplementary file 1 [file Data_Sheet_1.ZIP › Table S9 gall bladder disease cost questionnaire.docx]

Table S9 Questionnaire for medical cost of gall bladder disease per patient per year

|  | Unit cost | Weight | Frequency | Final cost |
| --- | --- | --- | --- | --- |
| **Diagnosis** |  |  |  |  |
| GP |  |  |  |  |
| Specialist physician visit |  |  |  |  |
| **Hospitalization** |  |  |  |  |
| Admission |  |  |  |  |
| IV antibiotics |  |  |  |  |
| anti-inflammatory |  |  |  |  |
| Proton pump inhibitors |  |  |  |  |
| IV fluids |  |  |  |  |
| **Imaging** |  |  |  |  |
| Ultrasound |  |  |  |  |
| MRCP (magnetic resonance cholangiopancreatography) |  |  |  |  |
| ERCP (endoscopic retrograde cholangiopancreatography) |  |  |  |  |
| CT abdomen/Pelvis |  |  |  |  |
| **Labs** |  |  |  |  |
| CBC |  |  |  |  |
| Renal functions (Urea and creatinine) |  |  |  |  |
| AST |  |  |  |  |
| ALT |  |  |  |  |
| Alkaline phosphatase |  |  |  |  |
| Fasting blood |  |  |  |  |
| PT |  |  |  |  |
| PTT |  |  |  |  |
| B and C virology |  |  |  |  |
| **Management** |  |  |  |  |
| **a)** Asymptomatic gallbladder stones |  |  |  |  |
| **b)** Symptomatic gallbladder stones |  |  |  |  |
| Laparoscopic cholecystectomy |  |  |  |  |
| Open cholecystectomy |  |  |  |  |
| **c) Conservative management** |  |  |  |  |
| **d)Common bile duct stones** |  |  |  |  |
| ERCP |  |  |  |  |
| Laparoscopic cholecystectomy |  |  |  |  |
| Total cost |  | | | |
